# PE38-based gene therapy of HER2-positive breast cancer stem cells via VHH-redirected polyamidoamine dendrimers

**DOI:** 10.1038/s41598-021-93972-5

**Published:** 2021-07-30

**Authors:** Cobra Moradian, Fatemeh Rahbarizadeh

**Affiliations:** Department of Medical Biotechnology, Faculty of Medical Sciences, Tarbiat Modares University, Tehran, Iran

**Keywords:** Biotechnology, Cancer, Stem cells, Nanoscience and technology

## Abstract

Breast cancer stem cells (BCSCs) resist conventional treatments and cause tumor recurrence. Almost 25% of breast cancers overexpress human epidermal growth factor receptor-2 (HER2). Here we developed a novel multi-targeted nanosystem to specifically eradicate HER2^+^ BCSCs. Plasmids containing CXCR1 promoter, PE38 toxin, and 5′UTR of the basic fibroblast growth factor-2 (bFGF 5'UTR) were constructed. Polyamidoamine (PAMAM) dendrimers functionalized with anti-HER2 VHHs were used for plasmid delivery. Stem cell proportion of MDA-MB-231, MDA-MB-231/HER2^+^ and MCF-10A were evaluated by mammosphere formation assay. Hanging drop technique was used to produce spheroids. The uptake, gene expression, and killing efficacy of the multi-targeted nanosystem were evaluated in both monolayer and spheroid culture. MDA-MB-231/HER2^+^ had higher ability to form mammosphere compared to MCF-10A. Our multi-targeted nanosystem efficiently inhibited the mammosphere formation of MDA-MB-231 and MDA-MB-231/HER2^+^ cells, while it was unable to prevent the mammosphere formation of MCF-10A. In the hanging drop culture, MDA-MB-231/HER^+^ generated compact well-rounded spheroids, while MCF-10A failed to form compact cellular masses. The multi-targeted nanosystem showed much better uptake, higher PE38 expression, and subsequent cell death in MDA-MB-231/HER2^+^ compared to MCF-10A. However, the efficacy of our targeted toxin gene therapy was lower in MDA-MB-231/HER2^+^ spheroids compared with that in the monolayer culture. the combination of the cell surface, transcriptional, and translational targeting increased the stringency of the treatment.

## Introduction

Breast cancer stem cells (BCSCs) are a subpopulation of breast cancer cells with self-renewal and differentiation capacity. Since BCSCs resist conventional treatments and cause tumor recurrence, targeting them may lead to the development of more effective cancer treatment modalities^1^. BCSCs survive and generate mammosphere in low-adherence serum-free cultures, whereas all the other cell types will undergo anoikis. Each mammosphere represents on average one stem cell of the parental population^2^. Based on this feature, we used mammosphere formation assay to determine the proportion of BCSCs in MDA-MB-231 and MDA-MB-231/HER^+^ populations as well as BCSC-like cells in pseudonormal MCF-10A and also to assess the effect of different dendriplexes on the cell lines MFE%. Targeted “*killer gene*” therapy is a promising approach for eradicating cancerous cells. Here, we utilized a CXCR1 promoter, PE38 toxin, and bFGF 5'UTR genetic construct delivery system composed of polyamidoamine (PAMAM) dendrimers functionalized with an anti-HER2 VHH to target HER2^+^ BCSCs simultaneously on transcriptional, translational, and cell surface levels. PE38 is a mutated form of the *Pseudomonas* exotoxin A. This highly cytotoxic protein arrests protein synthesis by inactivating eukaryotic elongation factor-2 (eEF-2)^3^. To date, various studies have reported that the introduction of PE38 rapidly kills the host cells^4,5^ In this research, we utilized CXCR1 promoter to restrict PE38 transcription to BCSCs and HER2^+^ breast cancer cells. CXCR1, a receptor for interleukin-8, is overexpressed in the aforementioned cells^6,7^. With the aim of translational targeting, bFGF 5'UTR was used as an additional discriminatory element to the CXCR1 promoter to improve the stringency and specificity of the treatment. Translation of mRNAs with long and highly structured 5ʹUTRs is largely dependent on the unwinding activity of eukaryotic translation initiation factor 4E (eIF4E). eIF4E is rare in most cell types^8^. Nonetheless, it is overexpressed in tumorigenic cells and facilitates the translation of GC-rich 5ʹUTR of the bFGF^9^.

PAMAM dendrimers are attractive vehicles for gene delivery. They effectively condense nucleic acids and protect them from being degraded by nucleases^10^. To avoid off-target effects, dendrimers can be equipped with a variety of tumor-specific targeting agents such as peptides, aptamers, antibodies, and VHHs. VHH is the smallest antigen-binding domain of *Camelidae* heavy chain antibodies. Their unique characteristics including exquisite affinity and specificity against their targets and high stability and solubility as well as low immunogenicity make them ideal targeting agents for the development of cancer treatment modalities^11,12^. With the aim of cell surface targeting, we functionalized PAMAM dendrimers with anti-HER2 VHHs. Almost 25% of breast cancers overexpress HER2^13^. HER2 overexpression confers a higher rate of aggressive metastasis^14,15^. Normally, the MDA-MB-231 cell line does not express HER2. However, a high percentage of BCSCs in MDA-MB-231 cell line has been reported previously^16,17^. MDA-MB-231/HER2^+^ is a cellular model developed in our laboratory via lentiviral transduction^18^. Our aim was to establish a breast cancer cell line with HER2 overexpression and a high percentage of BCSC subpopulations to assess the effect of combined cell surface HER2-targeting and BCSC-specific transcriptional targeting of our multi-targeted nanosystem.

In this study, we exploited the potential of our multi-targeted nanosystem in both monolayer and spheroid culture. Unlike monolayer culture, three-dimensional (3D) spheroid culture recaptures many features of the real tumor microenvironment, such as cell–cell contact and cell–extracellular matrix (ECM) interactions as well as oxygen, nutrient, and signal gradients. Hanging drop is an efficient method for cultivating spheroids with uniform size and shape. We utilized the “hanging drop” technique to prepare 3D spheroids. One distinct advantage of this technique over other spheroid culture methods is that this method obviates the need for uncontrolled mechanical forces and interference from exogenous materials. In detail, cells accumulate at the bottom of the drop under the force of gravity, secrete their own matrix components, and self-assemble into spheroids. These features offer a great potential to study the effects of antitumor treatments^19^. Of note, this study is the first example of in situ transfection of spheroid in hanging drop culture.

## Materials and methods

### Materials

The plasmid pRB391 plasmid which encodes PE38 was a kind gift from Professor Ira Pastan (National Institutes of Health). PAMAM G5 dendrimer and 2, 4, 6-trinitrobenzene sulfonic acid (TNBSA) were purchased from Sigma-Aldrich (St Louis, MO, USA). Traut’s reagent (2-Iminothiolane-hydrochloride) was obtained from Pierce (Rockford, IL, USA). NHS-PEG3500-MAL was purchased from Jenkem Technology (Beijing, China). MDA-MB-231 cells were obtained from DSMZ (Braunschweig, Germany). MDA-MB-231/HER2^+^ is a cellular model of HER2 overexpression, developed in our laboratory via lentiviral transduction as described previously^18^. MCF-10A cells were purchased from ATCC (Manassas, VA). All other reagents used in this study were of analytical grade and bought from Sigma-Aldrich Co.

### Anti-HER2 VHH production, expression, and purification

The anti-HER2 VHH clone was isolated from a large VHH library from one-humped and two-humped immunized camels using phage display technique^20^. The nucleotide sequence was subcloned into PET28, expressed in *Escherichia coli* (*E. coli*), and purified using immobilized metal ion affinity chromatography (IMAC) column, as described previously^21^.

### Gene constructs

The methods of making the gene constructs have been described in detail previously^22^. Briefly, CMV promoter was amplified by PCR from pGL4.50 (Promega, WI). The PCR products were subcloned into the pGL4.14 vector (Promega) to make pGL4.14-CMV (pG-CM). For the cloning of the CXCR1 promoter, 1123 bp fragment surrounding the putative promoter region upstream of the CXCR1 gene was amplified by PCR from peripheral lymphocyte DNA. The PCR product was inserted into the upstream of the luciferase gene in pGL4.14 to make pGL4.14-CXCR1 (pG-CX). For the cloning of bFGF 5'UTR, 400 bp of 5' region of the human bFGF mRNA sequence (chromosome 4q26; NG_029067.1; Gene ID: 2247) was selected. This fragment, flanked by NheI and BglII sites, was synthesized by Life Technology (Invitrogen, CA, USA) and received in pMK-RQ and then it was subcloned into pG-CX to make pGL4.14-CXCR1-bFGF 5'UTR (pG-CX-bF). The PE38 gene was amplified from pRB391 by PCR. The PCR product was substituted for luciferase gene of pG-CM, pG-CX, and pG-CX-bF to make pGL4.14-CMV-PE38 (pG-CM-PE), pGL4.14-CXCR1-PE38 (pG-CX-PE) and pGL4.14-CXCR1-bFGF 5'UTR-PE38 (pG-CX-bF-PE), respectively. The constructs were purified using the endotoxin-free plasmid DNA purification kit (MACHEREY-NAGEL), validated by sequencing, and then used for cell transfections.

### Preparation of VHH-PEG-PAMAM and Trastuzumab-PEG-PAMAM conjugates

G5 PAMAM was reacted with NHS-PEG3500-Mal in 2 mL of degassed phosphate buffer (pH 7.5) at a ratio of 1:10 (mol/mol) under N2 atmosphere at room temperature with gentle shaking for 2 h. The removal of unreacted PEG molecules was carried out by ultrafiltration using Amicon ultrafilters (MWCO 10 kDa; Millipore, Schwalbach, Germany). The number of PEG groups introduced to PAMAM was determined based on TNBSA assay. For the conjugation of the anti-HER2 VHHs to maleimide at the distal end of the PEGylated PAMAM dendrimers, the anti-HER2 VHHs were first thiolated using Traut’s reagent. Traut’s reagent was added to the anti-HER2 VHHs in borate buffer (50 mM sodium borate, 0.1 M EDTA, *pH* 8.3) to produce a ratio of 1:10 (mol/mol) and the reaction was performed under N2 atmosphere at room temperature with gentle shaking for 1 h. The removal of the unbound Traut molecules and replacing the buffer with phosphate buffer were carried out by ultrafiltration using Amicon ultrafilters (MWCO 30 kDa). Moreover, the thiolated VHHs were added to the PEG-PAMAM to produce a ratio of 2:1 (mol/mol), and the reaction was performed under N2 atmosphere at room temperature with gentle shaking for 18 h. The final conjugates (VHH-PEG-PAMAM) were purified using Amicon ultrafilters (MWCO 50 kDa), lyophilized, and stored at − 80 °C. All of the procedures used for the preparaton of the Trastuzumab-PEG-PAMAM conjugates were the same as those of the VHH-PEG-PAMAM conjugates except that the molar ratio of Traut’s reagent to Trastuzumab was 20:1 and the removal of the unbound Traut molecules were carried out by Amicon ultrafilters with a bigger pore size (MWCO 100 kDa). From now on, PEG-PAMAM, VHH-PEG-PAMAM, and Trastuzumab-PEG-PAMAM will be referred to as PG-PAM, VHH-PG-PAM, and Tra-PG-PAM, respectively.

### Characterization of PG-PAM and VHH-PG-PAM conjugates

#### Measuring the primary amines of PAMAM and PEGylated PAMAM

The primary amine groups of PAMAM and PEGylated PAMAM were measured with a spectrophotometer after the reaction of the free amine groups with TNBSA, as described by Snyder et al.^23^. The standard curve was generated using glycine serial dilutions (data not shown). The quantity of the PEG groups coupled with PAMAM were calculated by the differences in the amounts of primary amines on PAMAM and PEGylated PAMAM.

#### Characterization of PG-PAM by FTIR

To verify the PEGylation process, 1 mg of unmodified G5 PAMAM and the PG-PAM conjugates were analyzed by FTIR (Perkin Elmer instrument) at a resolution of 4.0 cm^−1^ via the KBr pellet method.

#### Characterization of the VHH-PG-PAM conjugates by H-NMR

The VHH-PG-PAM conjugates were characterized by nuclear magnetic resonance spectroscopy (NMR) using Bruker 400 MHz Avance II + H-NMR spectrometer (Bruker, Rheinstetten, Germany) in deuterium water (D_2_O).

### Dendriplex formation

Plasmid DNA and dendrimer solutions were prepared in HEPES-buffered glucose (HBG; HEPES 20 mM, Glucose 5% w/w, pH 7.4). Dendriplexes were prepared by mixing equal volumes of plasmid DNA solutions with PAMAM, PG-PAM, VHH-PG-PAM, or Tra-PG-PAM solutions at the N/P ratio of 10. The mixtures were vortexed for 30 s and left for 30 min at room temperature. Table 1 lists the prepared nanoparticle/gene construct complexes and the corresponding abbreviations.

**Table 1:** The list of prepared nanoparticle/gene construct complexes and their abbreviated name.

Test	Nanoparticle/gene construct complexes	Abbreviation
Cellular uptake	PAMAM/pEGFPN1 PEG-PAMAM/pEGFPN1 VHH-PEG-PAMAM/pEGFPN1 Trastuzumab-PEG-PAMAM/pEGFPN1	PAM/GFP PG-PAM/GFP VHH-PG-PAM/GFP Tra-PG-PAM/GFP
PE38 mRNA expression and PE38 cytotoxicity	PAMAM/pGL4.14 PAMAM/pGL4.14-CMV-PE38 VHH-PEG-PAMAM/pGL4.14-CMV-PE38 VHH-PEG-PAMAM/pGL4.14-CXCR1-PE38 VHH-PEG-PAMAM/pGL4.14-CXCR1-bFGF 5'UTR-PE38	PAM/pGL4.14 PAM/pG-CM-PE VHH-PG-PAM/pG-CM-PE VHH-PG-PAM/pG-CX-PE VHH-PG-PAM/pG-CX-bF-PE

### Dendriplex characterization

#### Gel retardation assay

The binding of the pG-CX-bF-PE to PAMAM, PG-PAM and VHH-PG-PAM were corroborated by agarose gel retardation assay. PAMAM or its conjugates were complexed with 1 μg plasmid at the N/P ratio of 10, and an amount of 1 μg free plasmid as a control, were run in 1% agarose gel (prepared in 1 M Tris–acetate-EDTA (TAE) buffer solution and stained simultaneously with Ethidium Bromide and Bromophenol Blue). Electrophoresis was performed at 100 constant volts for 30 min.

#### Dynamic light scattering and zeta potential measurements

PAM/pG-CX-bF-PE, PG-PAM/pG-CX-bF-PE, and VHH-PG-PAM/pG-CX-bF-PE dendriplexes were prepared according to the method mentioned in Dendriplex formation section and diluted by 800 μL of Milli-Q water. Their size and surface charge were measured by Zetasizer Nano ZS (Malvern Instruments, Malvern, UK).

#### Atomic force microscopy

The shapes and particle sizes of VHH–PG–PAM/pG-CX-bF-PE were analyzed using atomic force microscopy. Dendriplexes were prepared using VHH-PG-PAMAM complexed with pG-CX-bF-PE at an N/P ratio of 10 and incubated for 15 min at RT. The dendriplex solution was then diluted (1:20) with MilliQ-water. After that, a volume of 5 µL of the solution was placed on a freshly cleaved untreated mica surface and allowed to stick for 5 min. Then, the excess of the sample was removed by careful absorption onto filter paper and the mica surface were further dried under a gentle stream of air at RT. Sample were examined with a JPK AFM (JPK Instruments Co., Germany) using contact mode, HYDRA6V-100N cantilever with pyramidal shape tip, force constant of 0.292 N/m, and resonance frequency of 66 kHz.

#### Transmission electron microscopy

Transmission electron microscopy (TEM) images were obtained from CEM 902A ZEISS (Jena, Germany) transmission electron microscope with an accelerating voltage of 80 kV to investigate the size and morphology of VHH-PG-PAM/pG-CX-bF-PE at an N/P ratio of 10.

### Flow cytometry analysis to determine the percentage of HER2-expressing cells

Flow cytometry was used to evaluate the expression of HER2 on MCF10A, MDA-MB-231, and MDA-MB-231/HER2^+^. To do this, 70%-80% confluent cells were washed twice with phosphate-buffered saline (PBS) and then harvested with 0.05% trypsin (Thermo Fisher Scientific, USA). The detached cells were pelleted and re-suspended in PBS supplemented with 0.5% fetal bovine serum (1 × 10^6^ cells/50 μL). FITC-conjugated anti-ErbB2 antibodies (Abcam, Cambridge, UK) were added to the cell suspension and incubated at 4 °C in the dark for 30 min. Isotype control cells were incubated with FITC-conjugated mouse IgG (Abcam). The labeled cells were analyzed on a FACS Aria II Calibur (BD Biosciences). Data were analyzed with the Flowjo software version 7.2.4 (Tree Star Inc).

### Mammosphere cultivation

Mammospheres were generated from 2 × 10^4^ single cells seeded in 6-well tissue culture plates which were coated with 1.5% agarose and contained 2 mL DMEM/F12 (Gibco) without serum, supplemented with B27 (1:50, Invitrogen), 5 mg/mL insulin (Sigma), 20 ng/mL bFGF (R&D), and 20 ng/mL EGF (R&D). The cultures were incubated in a humidified atmosphere (5% CO_2_) at 37 °C for 3 days without any disturbance. After 3 days, 400 µL of fresh media was added to each well (without removing the old media). In the next step, the mammospheres were imaged on day 7. Moreover, the sizes and number of the mammospheres were quantitated using ImageJ software (NIH, USA) and mammosphere formation efficiency (MFE) was calculated as the number of mammospheres per well (diameter > 50 μm) divided by the original number of single cells seeded per well × 100. Experiments were performed in duplicates.

### Monolayer cell culture

MDA-MB-231 and MDA-MB-231/HER2^+^ were maintained in RPMI-1640 medium with 10% (v/v) FBS and penicillin/streptomycin. MCF10A were grown in DMEM-F12 medium (Invitrogen) containing Hydrocortisone (0.5 µg/mL), insulin (10 µg/mL), EGF (20 ng/mL), horse serum 5% (v/v) (Gibco), and penicillin/streptomycin. All cell lines were maintained in a humidified incubator (5% CO_2_) at 37 °C.

### Monolayer cell transfection

For cell transfection, 6 × 10^4^ cells/well were seeded and incubated until they reached ∼ 80% confluency. Immediately before the transfection process, the complete medium was removed, cells were washed with PBS and then fresh medium without serum and antibiotics was added to each well. Moreover, the dendriplexes were prepared freshly and prior to use and then 100 μL of the dendriplex solution at a final transfection DNA concentration of 2 µg/mL^−1^ was applied to the cultured cells. After 6 h of incubation, the transfection solution was removed, the cells were washed with PBS, and then cell-specific complete medium was added and the culture was further incubated for the time period required for each experiment**.**

### Hanging drop culture

MCF-10A and MDA-MB-231/HER^+^ cell lines were cultured as spheroids in their appropriate media by the hanging drop method. In detail, the cells were dissociated from the monolayer cell culture, counted, and resuspended in complete medium at a concentration of 3 × 10^4^ cells/mL. 13 µL aliquots of the suspension (containing 1000 cells each) were deposited on the underside of a 10 cm petri dish lid. The lid was then inverted over the dish filled with 10 mL of PBS (as a method to keep the cells hydrated). The dish was maintained at 37 °C in a humidified incubator (5% CO_2_) for 7 days. The growth media were exchanged every other day by up-righting the lid, taking 10 µL media from each drop, and adding 14 µL fresh media into it. The cells were imaged daily and the formed spheroids’ size (average of the major and minor axis length) was measured using the NIH ImageJ software.

### Spheroid transfection

On the seventh day following spheroid formation, the complete medium was replaced with PBS and subsequently with fresh medium without serum and antibiotics using the sequential pipetting method. The dendriplexes were prepared freshly prior to use and were carefully introduced to each of the spheroids by taking 14 µL media from and adding 14 µL of dendriplex solution to them. After 6 h of incubation, the medium was replaced with the complete medium using the sequential pipetting method. The hanging drop culture was further incubated for the time period required for each experiment. Then, the transfected spheroids were collected and dissociated to single cells by pipetting through a 200 µL pipet tip. In the next step, the obtained single cells were used for cellular uptake, PE38 mRNA expression, or cytotoxicity assays as described in subsequent Methods sections. For each assay, the experiments were performed in triplicate and 20 spheroids were analyzed per condition.

### Cellular uptake

To evaluate the gene transfection efficiency of the dendrimeric constructs in different cell lines, the dendriplexes of pEGFPN1 and PAMAM, PEG-PAMAM, VHH-PEG-PAMAM, or Trastuzumab-PEG-PAMAM were prepared as described in the “Dendriplex formation” section. Next, MCF-10A, MDA-MB-231, MDA-MB-231/HER2^+^, and MDA-MB-231/HER2^+^ spheroids were transfected with the aforementioned dendriplexes. Forty-eight hours after transfection, the expression of GFP was analyzed using a fluorescence microscope (Carl Zeiss, NY) and flow cytometry (FACS Aria II Calibur, BD Biosciences). The data were analyzed by Flowjo software version 7.2.4 (Tree Star Inc).

### PE38 mRNA expression analysis

Sixteen hours after the transfection process with different dendriplexes, the cells were collected, the total RNA was isolated with the High Pure RNA Isolation kit (Roche), and subsequently reverse transcribed using M-MuLV reverse transcriptase and oligo-dT (Fermentas). Next, real-time PCR was performed by Rotorgene 3000 series PCR machine (Corbett Research, San Francisco, USA) using RealQ Plus 2 × Master Mix Green (Amplicon, Denmark). All mRNA quantification data were normalized to β-actin. Also, the primers for amplifying PE38 and β-actin were as follows: PE38 for: 5ʹ AGGACCTCGACGCGATCTG, PE38 Rev: 5ʹ TCAGGCTGGTGCGGTAGAAG, β-actin for: 5ʹ TCC CTGGAGAAGAGCTACG, and β-actin Rev: 5ʹ GTAGTTTCGTGGATGCCACA. Moreover, the relative PE38 mRNA expression in different samples were calculated using Pfaffl method and the experiments were repeated three times.

### Cytotoxicity study

To evaluate PE38 cytotoxicity, the cell viability of the transfected cells vs. non-transfected cells was determined by MTT assay. Briefly, the cells were seeded and transfected as described for monolayer or spheroid culture. 6 h after transfection with different dendriplexes, the transfection solution was removed and then the cells were washed with PBS and later complete medium was added to each well. After 48 h of incubation, the complete medium was replaced by fresh medium without serum and antibiotics and then 1 mg/mL MTT reagent (3-(4,5-dimethylthiazol-2-yl)-2,5-diphenyltetrazolium bromide; Sigma) was added to each well according to the manufacturer’s protocols. The assay was repeated three times.

### Inhibition of mammosphere formation

We analyzed the impact of PAM/pG-CM-PE, VHH-PG-PAM/pG-CM-PE, VHH-PG-PAM/pG-CX-PE, and VHH-PG-PAM/pG-CX-bF-PE on the formation of MCF-10A, MDA-MB-231, and MDA-MB-231/HER2^+^ mammospheres by addition of these dendriplexes (100 μL of dendriplex solution at a final transfection DNA concentration of 2 µg/mL) to the culture medium either at seeding or after five days of growth in mammosphere culture conditions as described in the mammosphere cultivation section. After 6 h of incubation, the transfection solution was replaced with fresh medium. The experiments were done in duplicates.

### Statistical analyses

The statistical analyses were performed using GraphPad PRISM v.6.01 (GraphPad Software Inc, CA, USA). All data subjected to statistical analysis were obtained from at least three parallel experiments. Unpaired Student's t-test was used to determine significant differences between each group. Two-way ANOVA with Tukey’s multiple comparison post hoc test was used to determine significant differences between different groups. A *p*-value ≤ 0.05 was considered statistically significant.

## Results and discussion

### Characterization of PG-PAM and VHH-PG-PAM conjugates

#### Measuring primary amines of PAMAM and PEGylated PAMAM

TNBSA assay confirmed that PEGylation of dendrimers with molar ratios of 10 yielded conjugates containing an average of 19 PEG chains per PAMAM molecule.

#### Characterization of PG-PAM by FTIR

**Figure 1:**
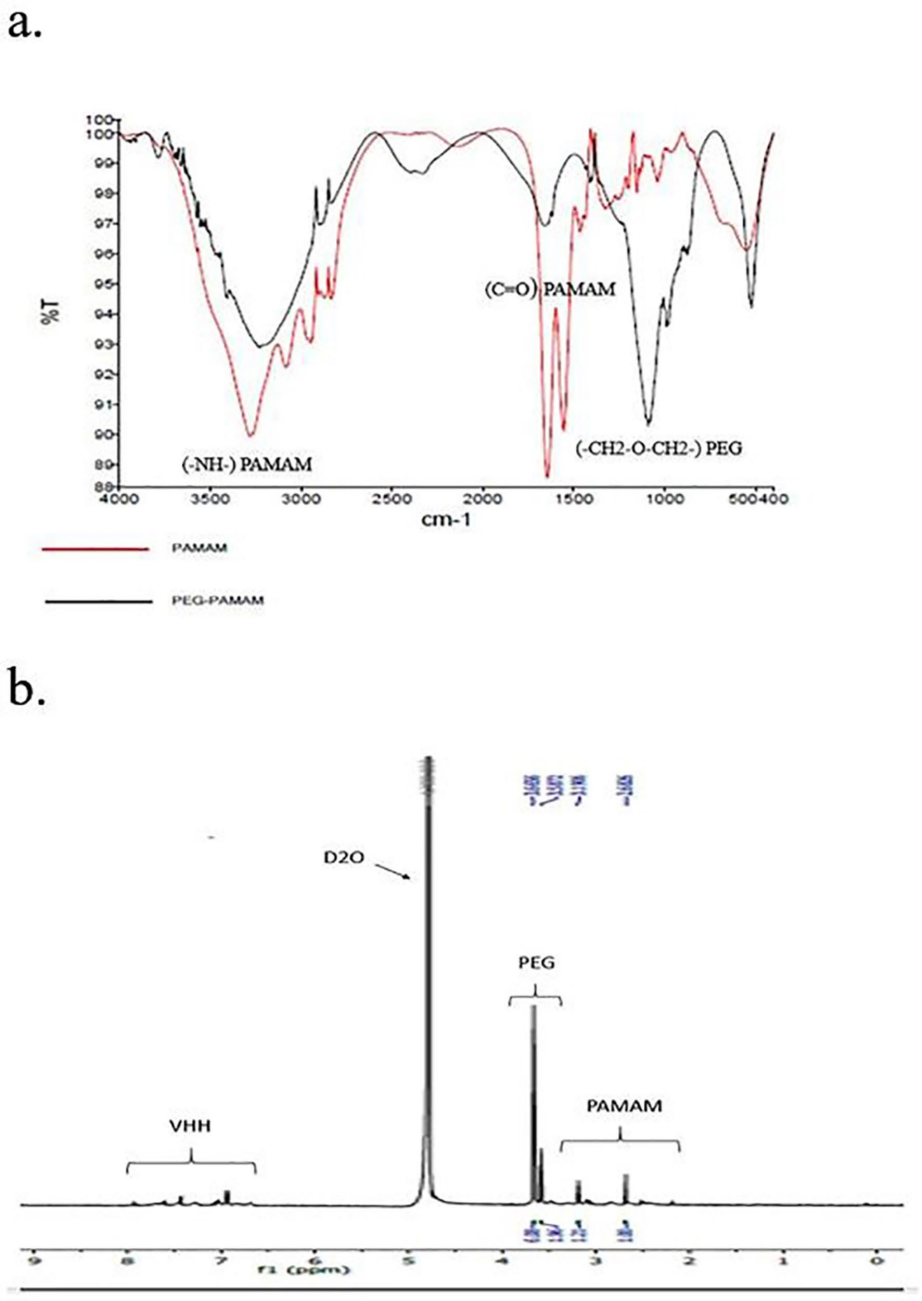
(**a**) FTIR spectra of PAMAM and PG-PAM. (**b**) H-NMR data of VHH-PG-PAM.

The formation of the PG-PAM conjugates was further confirmed by FTIR spectra. In FTIR spectra (Fig. 1a), the peak at 3300 cm^–1^ due to –NH– groups of PAMAM stretching vibration is evident. The characteristic peaks of methyl C=O stretching of carbonyl groups of PAMAM internal amides or PG–PAM amide bonds is found at 1650 cm^–1^. Besides, the peak at 1110 cm^–1^ due to stretching vibration of –CH2–O–CH2– etheric bonds of PEG molecules, indicating that PEG chains are successfully attached to PAMAM dendrimers.

#### Characterization of VHH-PG-PAM conjugates by H-NMR

The successful synthesis of VHH-PG-PAM was confirmed by H-NMR (Fig. 1b). The PAMAM dendrimer showed multiple peaks between 2.3 and 3.3 ppm such as δ 2.2–2.5 (–CH2CH2CONH–); δ 2.6 (–CH2CH2N <) and δ 3.2–3.3 (–CONHCH2CH2–). The peaks between 3.5 and 3.6 ppm belong to the (–CH2CH2O–) units of PEG chains. The VHH showed peaks between 7 and 8 ppm related to the benzene rings of aromatic amino acids in VHH protein sequence, indicating the successful conjugation of the VHH and PAMAM.

### Dendriplex characterization

#### Gel retardation assay

**Figure 2:**
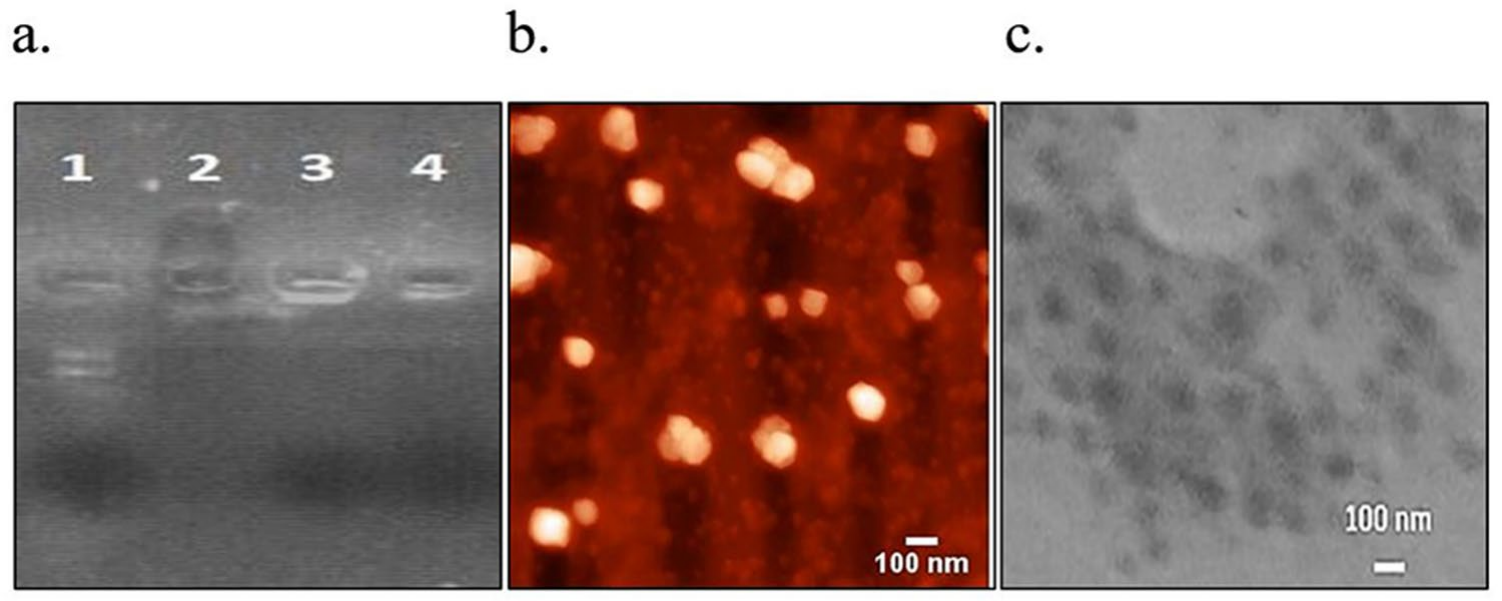
(**a**) Plasmid mobility retardation assay by 1% agarose gel. Lane 1, Pure pG-CX-bF-PE plasmid; Lane 2, PAM/pG-CX-bF-PE; Lane 3, PG-PAM/pG-CX-bF-PE; Lane 4, VHH-PG-PAM/pG-CX-bF-PE. (**b**) AFM image of VHH-PG-PAM/pG-CX-bF-PE. (**c**) TEM image of VHH-PG-PAM/pG-CX-bF-PE.

Agarose electrophoretic mobility retardation assay was performed using PAM/pG-CX-bF-PE, PG-PAM/pG-CX-bF-PE and VHH-PG-PAM/pG-CX-bF-PE, at N/P ratio of 10. The purified pG-CX-bF-PE plasmid moved toward anode (Fig. 2a, lane 1). In contrast, Fig. 2a, lanes 2 shows that, not only PAMAM reversed the plasmid electrophoresis pattern, but also the negatively charged Bromophenol Blue dye were also electrophoresed to the opposite direction, cathode. This result suggests that the overall charge of PAM/pG-CX-bF-PE were comparatively positive. Figure 2a, lanes 3 and 4 show that, PG-PAM and VHH-PG-PAM interacted sufficiently with the pG-CX-bF-PE and neutralized its negative charge so the plasmid were retarded in the well( Full-length gel image is provided in supplementary Fig. S1).

#### Dynamic light scattering and zeta potential measurements

The size and zeta potential of the dendriplexes at N/P ratio of 10 were measured by zetasizer Nano ZS. According to results presented in Table 2, all the dendriplexes showed the appropriate size and charge density. Low polydispersity indexes indicate the formation of homogeneous and aggregate free dendriplexes. It seems that by conjugating PEG and VHH to the PAMAM dendrimers, the size of dendriplexes increased, while the zeta potential was decreased. These findings imply that both PEG and VHH were successfully attached to the dendrimers and shielded the positive charges of the free amines on the PAMAM surface. However, the size of dendriplexes ranged between 105.6 and 154.7 nm and the zeta potential ranged between 26.8 and 5.2 mV which are considered to be appropriate for endocytosis.

**Table 2:** Average size and zeta-potential of the dendriplexes at N/P ratio of 10.

Dendriplex	PDI	Size (nm)	Zeta potential (mV)
PAM/pG-CX-bF-PE	0.16	105.6	26.8
PG-PAM/pG-CX-bF-PE	0.24	127.4	11.4
VHH-PG-PAM/pG-CX-bF-PE	0.21	154.7	5.2

#### Atomic force microscopy

The morphology of VHH-PG-PAM/pG-CX-bF-PE at an N/P ratio of 10, were observed by AFM (Fig. 2b). The result obtained revealed that the dendriplexes formed spherical particles with an average diameter of 121 ± 2 nm and a narrow size distribution, falling within the optimum size requirements (100–200 nm) for efficient cellular endocytosis.

#### Transmission electron microscopy

Transmission electron microscopy was performed to further investigate the size and morphology of VHH-PG-PAM/pG-CX-bF-PE dendriplexes. TEM result was in accordance with the AFM data, showing spherical structures with an average particle size of 118 ± 7 nm (Fig. 2c). However, the particle size visualized by TEM and AFM were smaller than those determined by DLS. The most probable explanation would be that TEM and AFM determine the dry particle size, whereas DLS reflects the hydrodynamic size.

### Percentage of HER2 expressing cells

**Figure 3:**
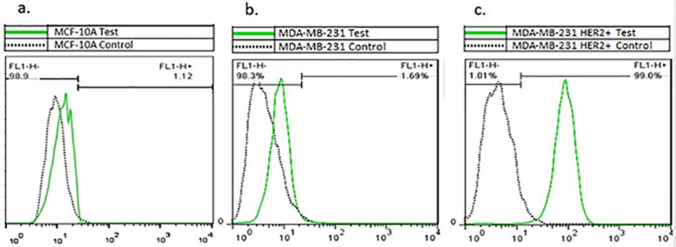
(**a**) Flow cytometric analysis of MCF-10A. (**b**) MDA-MB-231 and (**c**) MDA-MB-231/HER2^+^. Cells were stained with FITC-conjugated anti-ErbB2 antibody and analyzed by flow cytometry (BD Biosciences). The experiments were repeated in duplicate. Data were analyzed with the Flowjo software (Tree Star Inc).

MCF10A, MDA-MB-231 and MDA-MB-231/HER2^+^ cell lines were analyzed using flow cytometry for HER2 expression. As illustrated in Fig. 3a, b, MCF10A and MDA-MB-231 demonstrated a weakly positive *HER2* expression (1.12% and 1.89% respectively). Whereas, 99.00% of MDA-MB-231/HER2^+^ cells expressed *HER2* cell surface marker (Fig. 3c).

### There are discernible differences in BCSC pools between MCF-10A and MDA-MB-231/HER2^+^

**Figure 4:**
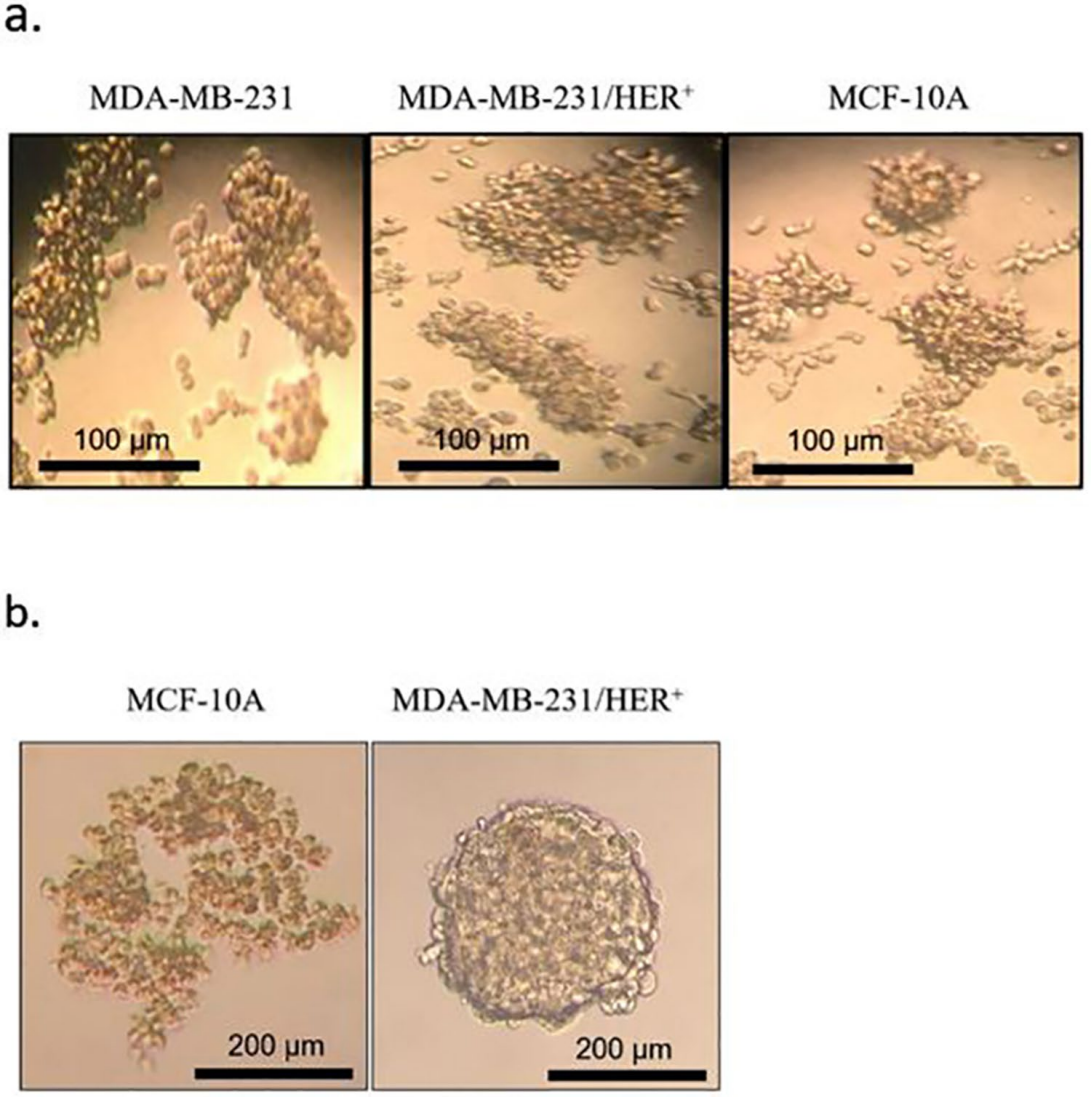
(**a**) Optical microscope images showing the MDA-MB-231, MDA-MB-231/HER^+^ and MCF-10A cell lines cultivated in non-adherent mammosphere culture for 7 days. (**b**) Images of MCF-10A and MDA-MB-231/HER^+^ cells incubated in hanging drop culture for 24 h. 1000 cells/drop were used as seeding concentration Images were analyzed with ImageJ software (NIH).

Mammosphere assay was used to evaluate the stem property of the cell lines. According to Fig. 4a, both cell lines had the ability to form compact mammospheres. MCF-10A generated small round spheres, whereas MDA-MB-231 and MDA-MB-231/HER2^+^ formed large irregular shapes. However, after one week under mammosphere culture, the MFE of MDA-MB-231 and MDA-MB-231/HER2^+^ were almost the same(1.7 ± 0.87% and 1.8 ± 0.43%, respectively). Nonetheless, clear variations in the MFE of the two mentioned cell lines with MCF-10A (0.5 ± 0.11%) were observed, representing higher BCSC pool in MDA-MB-231 and MDA-MB-231/HER2^+^.

Mammosphere formation assay identifies BCSCs based on their resistance to anoikis in non-adherent/serum-free conditions. This technique is considered to be a reliable technique since conventional BCSC specific markers occasionally vary infinitely from one breast cancer cell to another^24^ The data of our previous work also agreed with this concept^22^, thus in the present study we based our work on the ability of BCSCs to form mammospheres."

### Morphology of spheroids in hanging drops

**Figure 5:**
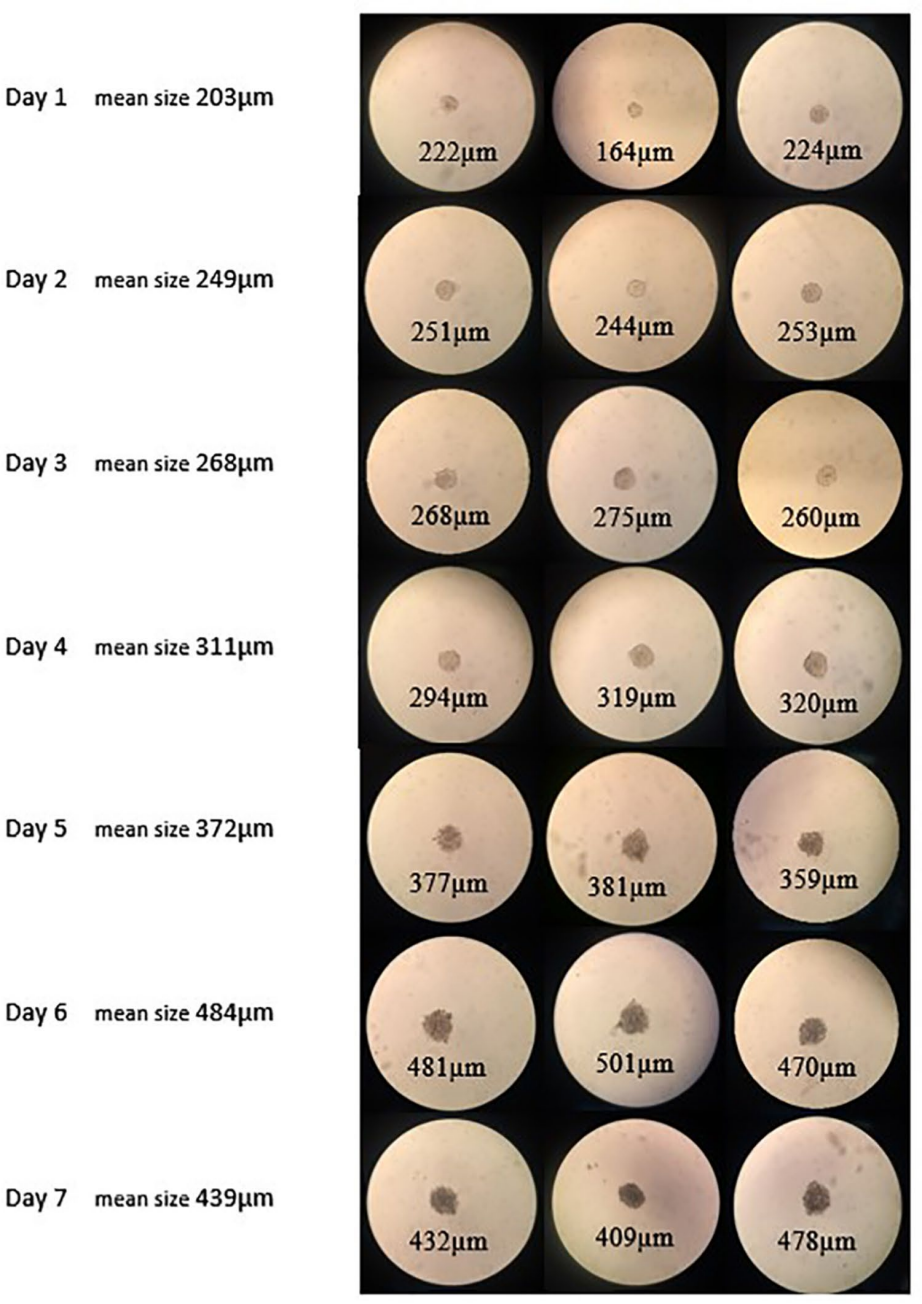
Images of MDA-MB-231/HER^+^ spheroids taken daily over a 7 days period in hanging drops. Images were analyzed with ImageJ software (NIH).

We utilized the hanging drop technique to generate spheroids with uniform shape and size. MCF-10A grown in hanging drop culture formed thoroughly loose aggregates exposing free areas between the cells. While MDA-MB-231/HER^+^ generated tightly compact well-rounded spheroids within 24 h (Fig. 4b). Based on these results, MCF-10A aggregates were excluded from further investigation and MDA-MB-231/HER^+^ spheroids were selected for subsequent studies. MDA-MB-231/HER^+^ spheroids were maintained in hanging drop culture for 7 days and imaged daily. As illustrated in Fig. 5, spheroids of different drops were consistent in both size and shape. On the first day spheroids measured approximately 203 μm. From first to sixth day the spheroids diameter increased slightly. On the sixth day their average size reached to 484 μm. However, by day 7 it decreased to 439 μm, indicating spheroids started to become denser. Several researchers suggest that spheroids of diameter ranging between 300 and 500 μm accurately mimic real tumors^25–27^. As it can be seen in Fig. 5, with increasing length of hanging drop culture, spheroids became a little darker in color, which is an indicative of gradual compactness^28^. On the seventh day following hanging drop culture, spheroids were transfected with the previously mentioned dendriplexes. It is noticeable that, spheroids were washed and transfected while they were in the drops, which to the best of our knowledge has not been previously accomplished. Next, the transfected spheroids were used to evaluate the discrimination of our treatment efficacy in MDA-MB-231/HER2^+^ monolayer and spheroid culture.

### Anti-HER2 VHH functionalization improved cellular uptake in the HER2 overexpressing cell line

**Figure 6:**
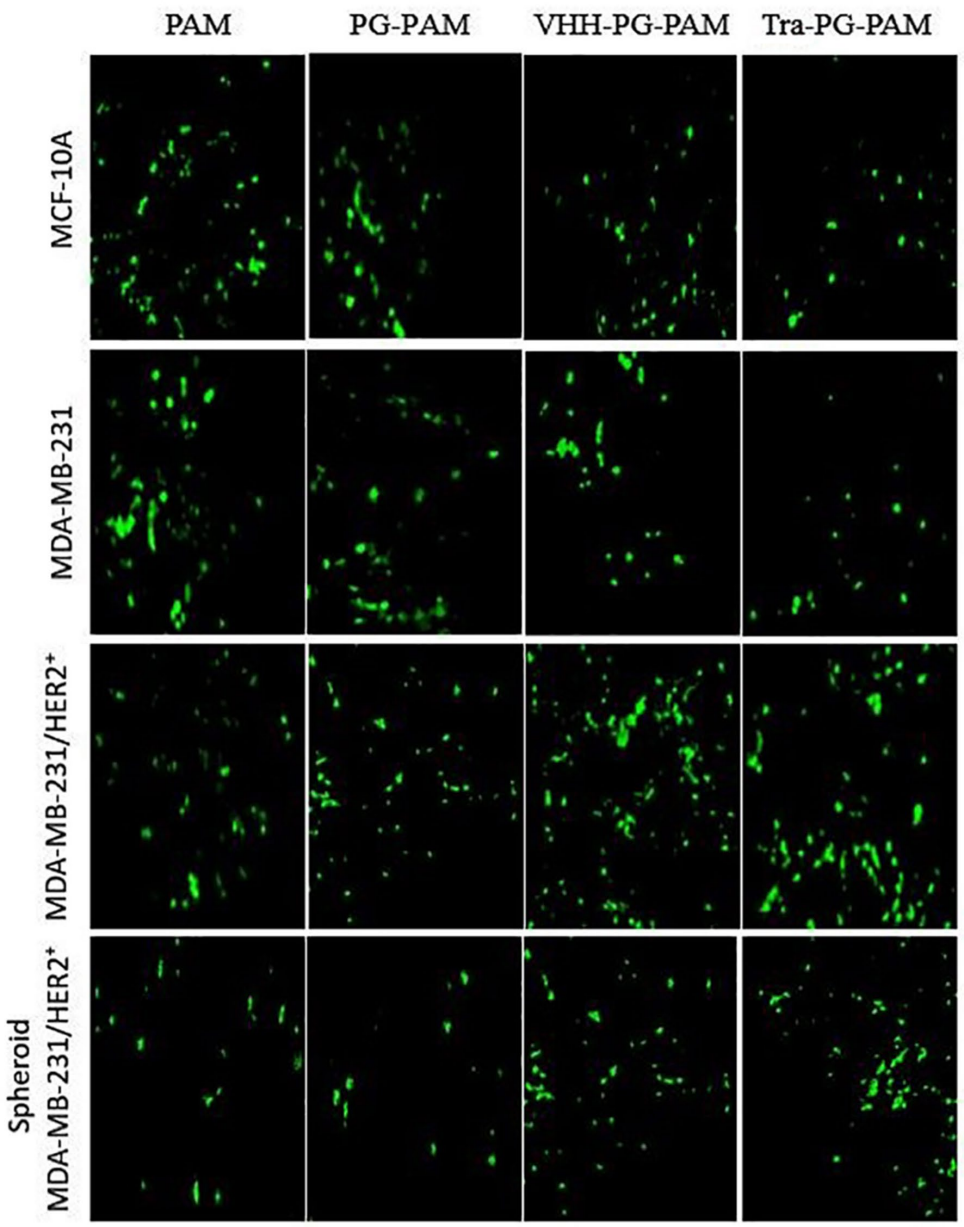
Fluorescence images of GFP-expressing cells. Cell lines were transfected with polyplexes complexed with pEGFPN1 at N/P ratio of 10. Images were taken 48 h post-transfection.

**Figure 7:**
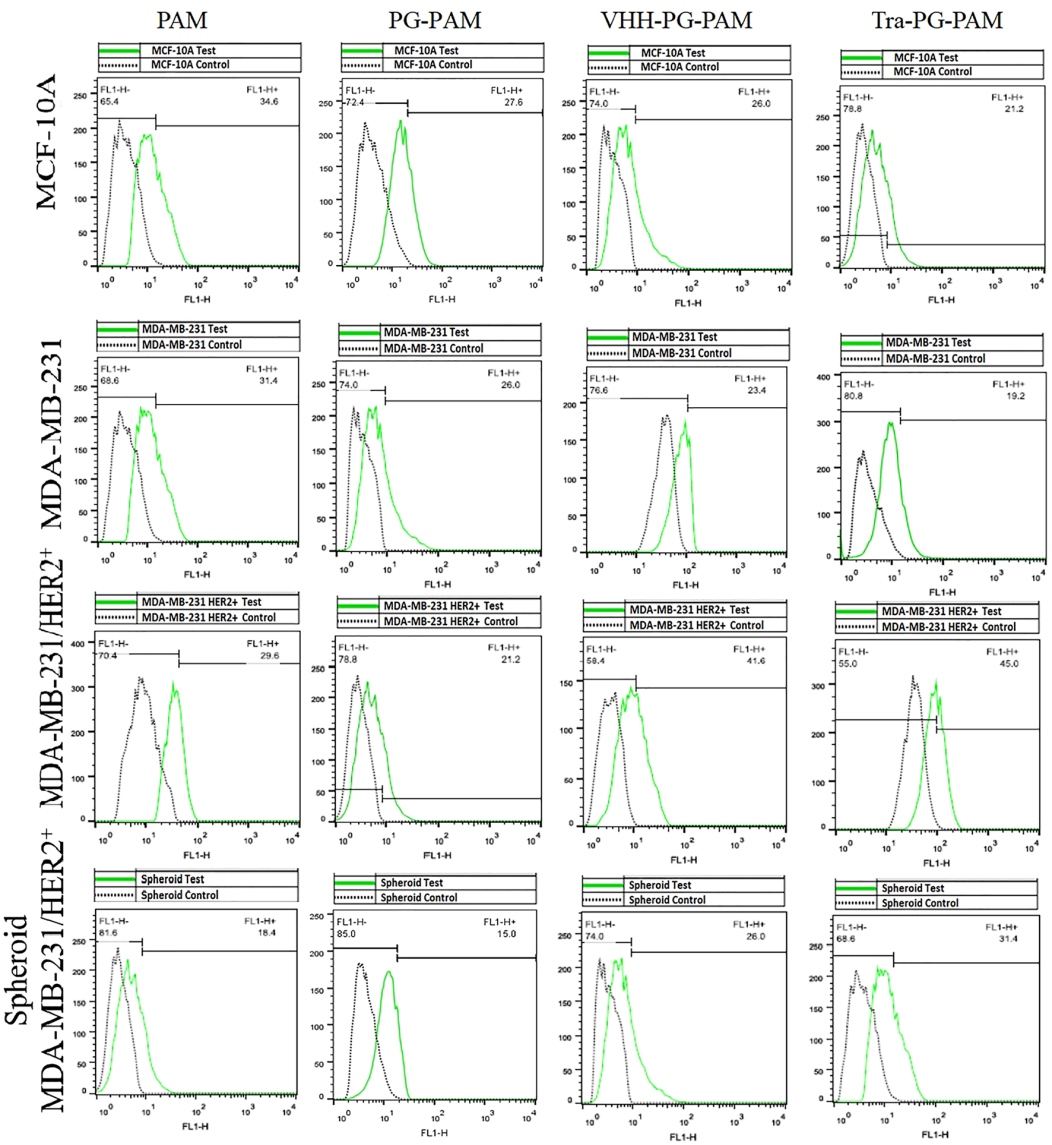
Flow cytometry results of GFP expressing cells. Cell lines were transfected with polyplexes complexed with pEGFPN1 at N/P ratio of 10. Percentages of GFP-expressing cells were measured 48 h post-transfection.

Fluorescence microscopy and flow cytometry was employed to compare the uptake efficiency of dendrimers and evaluate targeting efficiency of the anti-HER2 VHH. As it can be seen in Figs. 6 and 7, unmodified PAMAM transfection efficacy were comparable in all the three cell lines. However, PEGylation suppressed the cellular uptake to some extent. Similarly, Fant et al. reported that PEGylation of PAMAM dendrimers reduced its transfection efficacy^29^. In fact, PEGylation reduces the transfection efficacy of cationic dendrimers, yet increases their biocompatibility, probably by lowering their surface positive charge^30^. In MCF-10A and MDA-MB-231, VHH-PG-PAM, as well as Tra-PG-PAM, showed lower uptake as compared to PAM. In contrast, the transfection capability of these two targeted conjugates was significantly higher in MDA-MB-231/HER2^+^ (both monolayer and spheroids). However, differences between VHH-PG-PAM and Tra-PG-PAM transfection efficacy were negligible. Both conjugated and non-conjugated dendrimers were likely taken up by MCF-10A and MDA-MB-231 via non-specific electrostatic interactions with the cell surface. The unexpected lower transfection efficacy of VHH-PG-PAM, as well as Tra-PG-PAM, as compared to PAM in low/non-HER2 expressing MCF-10A and MDA-MB-231 cell lines might be attributed to the lower net positive charge and larger particle size of the conjugated dendrimers. Nevertheless, the superior transfection efficiency of the targeted dendrimers in MDA-MB-231/HER2^+^ was likely due to the HER2 receptor-mediated endocytosis rather than non-specific electrostatic interactions with the cell surface. Comparison of MDA-MB-231/HER2^+^ grown as monolayer vs spheroid revealed that internalization of all the dendriplexes were significantly lower in the spheroids. A possible reason for the lower uptake rate in spheroids might be the penetration resistance inherent in their structure. Spheroid is a three-dimensional (3D) model that closely resembles small avascular tumors and micrometastases in that they contain a proliferative outer shell, a relatively large zone of hypoxic quiescent cells and a relatively small necrotic area at the center. This 3D structure exhibit cell-to-cell and cell-to-matrix interactions as well as nutrient, *pH* and oxygen gradients. These characteristics make them proportional to penetration so that they are not efficiently transfected. This kind of resistance might not be observed when cells are cultured as monolayer^31,32^. In overall, VHH-PG-PAM gene delivery efficiency in the three cell lines followed the order of MDA-MB-231/HER2^+^ > MDA-MB-231/HER2^+^ spheroids > MCF-10A > MDA-MB-231. From these results, it can be concluded that the anti-HER2 VHH modified PAMAM can be considered as an efficient gene and drug carrier to target HER2^+^ breast cancer cells.

### Analysis of PE38 mRNA expression reveals efficient cell surface and transcriptional targeting

**Figure 8:**
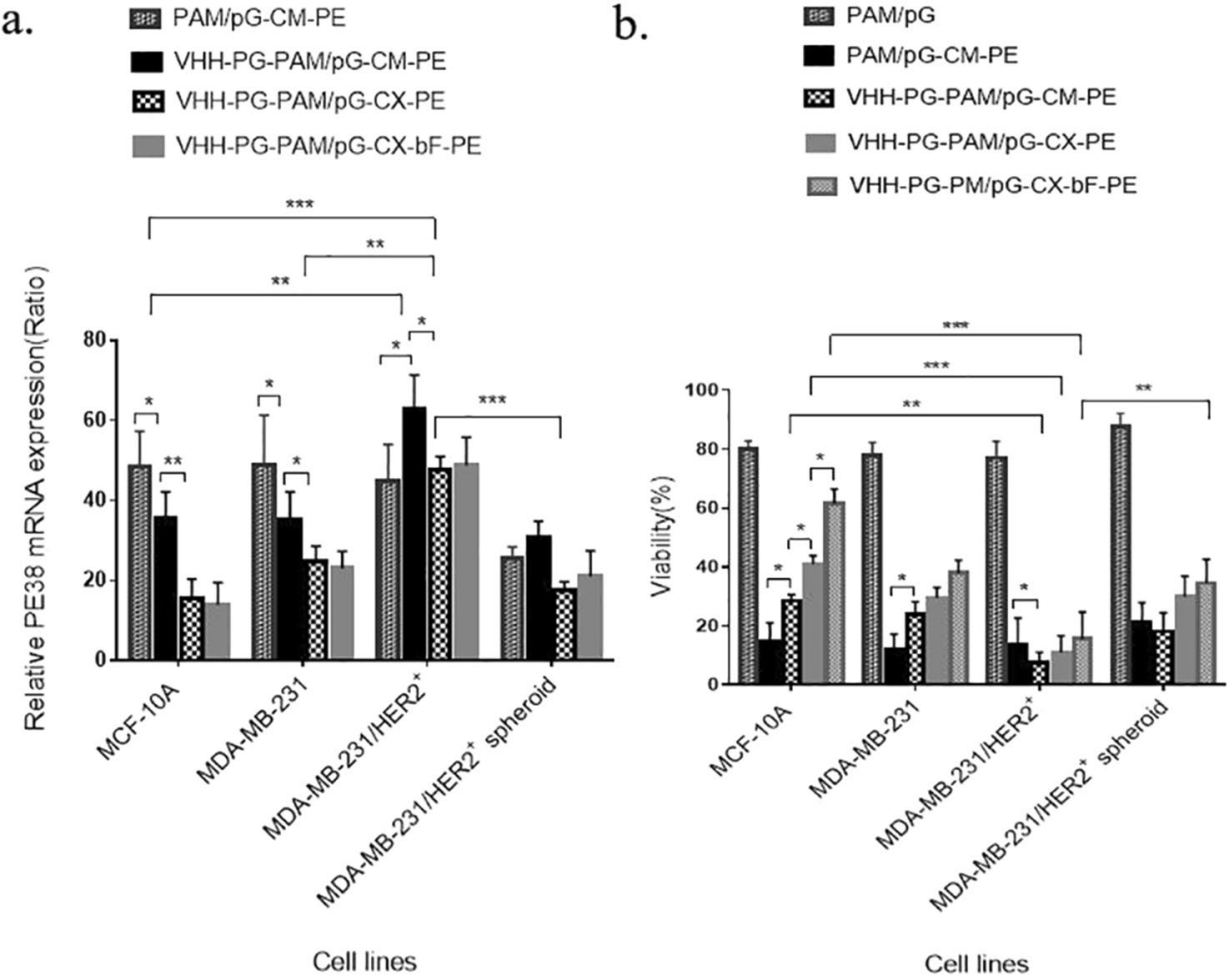
(**a**) PE38 gene expression on mRNA level in cells transfected with various constructs. All samples were normalized to mRNA levels for β-actin. (**b**) Cell viability of transfected cells vs. non-transfected cells determined by MTT assay. Each bar represents the average of a minimum of three independent transfections. Error bars represent the standard deviation. The statistical significance of the groups was calculated using t-test, two-way ANOVA and Tukey’s test for multiple comparisons. A *p*-value ≤ 0.05 was considered statistically significant (*p < 0.05; **p < 0.01; ***p < 0.001; ****p < 0.0001).

MCF-10A, MDA-MB-231, MDA-MB-231/HER2^+^ and MDA-MB-231/HER2^+^ spheroid were transfected with PAM/pG-CM-PE, VHH-PG-PAM/pG-CM-PE, VHH-PG-PAM/pG-CX-PE and VHH-PG-PAM/pG-CX-bF-PE dendriplexes and their respective PE38 mRNA expression pattern were analyzed by real-time PCR. First, we will discuss the targeting efficacy of the different dendriplexes in the three cell lines (cultured as monolayer) together, then MDA-MB-231/HER2^+^ monolayer will be compared with its spheroid. As illustrated in Fig. 8a, when transfected with PAM/pG-CM-PE (non-targeted), all the three cell lines expressed a high level of PE38 mRNA, with no significant differences among the cell lines (*p* = ns for all). As expected, the level of PE38 mRNA exhibited variation between cell lines when the targeted dendriplexes were used. VHH-PG-PAM/pG-CM-PE (targeted at cell surface level) treated cells showed equally lower expression of PE38 mRNA in both low/non-HER2 expressing cell lines (MCF-10A and MDA-MB-231). Whereas, the expression increased significantly in MDA-MB-231/HER2^+^ (*p* < 0.05 for all). These observations confirm that the anti-HER2 VHH conjugation led to higher penetration efficiency of PAMAM dendrimers in the HER2 expressing cell line. In case of VHH-PG-PAM/pG-CX-PE (targeted at cell surface and transcriptional level) treated cells as compared to VHH-PG-PAM/pG-CM-PE (targeted at cell surface level), the expression decreased in all the three cell lines, probably because CMV promoter is stronger than CXCR1promoter. However, the reduction were more significant in MCF-10A (*p* < 0.01) compared to BCSC rich MDA-MB-231 and MDA-MB-231/HER2^+^ (*p* < 0.05 for both), probably due to the CXCR1 specificity for BSCSs and HER2 expressing breast cancer cells^7^. In addition, among the three cell lines transfected with VHH-PG-PAM/pG-CX-PE, the highest PE38 mRNA expression belonged to the MDA-MB-231/HER2^+^, indicating both good cell surface targeting and efficient transcriptional targeting. There were no statistical differences in mRNA expression, between VHH-PG-PAM/pG-CX-PE and VHH-PG-PAM/pG-CX-bF-PE treated cells (*p* = ns for all), since bFGF 5′UTR modulate the gene expression specifically on translation level and not at the transcriptional level. Comparison of MDA-MB-231/HER2^+^ grown as monolayer vs. spheroid revealed that, MDA-MB-231/HER2^+^ spheroid followed the same expression pattern as its monolayer, upon all the treatments, except the mRNA expression level were significantly lower in spheroids (*p* < 0.001), probably due to the lower cellular uptake as demonstrated by fluorescence microscopy. The low uptake of therapeutic agents in solid tumors is one of the major obstacles to the successful gene or drug delivery.

### A combination of the cell surface, transcriptional and translational targeting, increased the stringency of the treatment

MCF-10A, MDA-MB-231, MDA-MB-231/HER2^+^ and MDA-MB-231/HER2^+^ spheroids were transfected with the PAM/pG, PAM/pG-CM-PE, VHH-PG-PAM/pG-CM-PE, VHH-PG-PAM/pG-CX-PE and VHH-PG-PAM/pG-CX-bF-PE dendriplexes. The cytotoxic effect of PE38 on the transfected cells was evaluated using MTT assay. First, we will discuss the cytotoxic effect of the different dendriplexes in the three cell lines (cultured as monolayer) together, then MDA-MB-231/HER2^+^ grown as monolayer and spheroid will be compared. PAM/pG (empty vector) was used to determine the toxicity of the transfection process and as shown in Fig. 8b, the viability was about 80% in the three cell lines. In contrast, following treatment with PE38 encoding vectors, extensive cell death was detected. This result reveals the clear cytotoxic potential of this toxin.

However, having transfected with PAM/pG-CM-PE (non-targeted), all the three cell lines exhibited about 13% viability. In the case of VHH-PG-PAM/pG-CM-PE(targeted on cell surface level), 28% and 24% cell viability for MCF-10A and MDA-MB-231 were detected, respectively; but it was 7% for MDA-MB-231/HER2^+^. These results suggest that VHH-PG-PAM/pG-CM-PE exerts potent cytotoxicity in HER2-overexpressing cells due to the specific cell surface targeting. VHH-PG-PAM/pG-CX-PE exhibited higher viability rate, as compared to VHH-PG-PAM/pG-CM-PE in all the cell lines, showing that CXCR1 promoter is weaker than the commonly used strong CMV promoter. Cell viability of MCF-10A and MDA-MB-231 transfected with the VHH-PG-PAM/pG-CX-PE (targeted on cell surface and transcriptional levels) were 41% and 29%, respectively, while in the MDA-MB-231/HER2^+^ it was 11%. However, VHH-PG-PAM/pG-CX-PE demonstrated more viability variation between the cell lines compared to the non-specific CMV promoter, and the lowest viability belonged to the MDA-MB-231/HER2^+^, indicating CXCR1 promoter specificity for BSCSs and HER2 expressing breast cancer cells. These results also demonstrate that, combination of the cell surface and transcriptional targeting, increased the stringency of the treatment. When transfected with VHH-PG-PAM/pG-CX-bF-PE (targeted on cell surface, transcriptional and translational levels), MCF-10A and MDA-MB-231 exhibited 61% and 38% viability, respectively. But MDA-MB-231/HER2^+^ showed 15% recovery of the cells. When comparing the effect of treatments with dendriplexes targeted on "cell surface", "cell surface and transcriptional" and "cell surface, transcriptional and translational" levels, as it can be seen in Fig. 8b, by the addition of each targeting element to the others, viability variation between different cell lines were increased. This result, indicates that the stringency of the treatment increases when these targeting elements are combined together. It is noticeable that, viability of VHH-PG-PAM/pG-CX-bF-PE treated groups had a tendency to increase as compared to that of VHH-PG-PAM/pG-CX-PE treated groups, probably because the highly structured, GC rich 5' UTR of the bFGF hinders efficient translation. Translation of mRNAs with highly structured bFGF 5′UTR is particularly dependent on the unwinding activity of eIF4E. Higher levels of eIF4E in breast cancer cell lines, relative to non-malignant MCF10A cells have been reported previously^33–35^. Thus, it can be concluded that overexpression of eIF4E in tumorigenic MDA-MB-231 and MDA-MB-231/HER2^+^ might be responsible for efficient toxin translation and subsequent massive cell death. Nonetheless, low levels of eIF4E in normal MCF-10A repressed toxin translation, resulting in elevated viability. Our study findings are consistent with other reports showing the efficient targeting efficacy of bFGF 5′UTR toward cancerous cells^9,33,36^.

Comparison of MDA-MB-231/HER2^+^ grown as monolayer vs spheroid revealed that MDA-MB-231/HER2^+^ spheroid followed the same viability pattern as its monolayer, upon all the treatments, except the viability were significantly higher upon all the treatments (*p* < 0.01). These results further validated those obtained by cellular uptake and real-time PCR experiments. Indeed, previous studies have demonstrated a differential response to compounds when cells grown as spheroid compared to monolayer^37,38^. A good illustration would be the study of Carver et al.^39^. They demonstrated that the delivery of oligonucleotides with Lipofectamine lipoplex and PEI polyplex was significantly attenuated in spheroid models compared to monolayer cultures. Further, their results showed that only cells located at the periphery of the spheroid received the oligonucleotides.

### Our multi-targeted nanosystem efficiently inhibited mammosphere formation of MDA-MB-231 and MDA-MB-231/HER2^+^ cells, while it was unable to prevent mammosphere formation of MCF-10A

To confirm the data obtained with MTT, we examined the effect of different dendriplexes, on the formation of mammospheres. The outcome revealed that, when the cells were transfected at the seeding step, the addition of PAM/pG-CM-PE resulted in the complete proliferation inhibition of all the three cell lines. Also, all of the dendriplexes strongly inhibited mammosphere formation of MDA-MB-231 and MDA-MB-231/HER2^+^. However, when they were transfected after five days growth in mammosphere culture conditions, the existing mammospheres were completely dissociated by Day 7. These results can be explained by the high cytotoxicity potential of PE38 protein. It is believed that expression of only one PE38 molecule efficiently kills the host cell l40 Interestingly, VHH-PG-PAM/pG-CM-PE, VHH-PG-PAM/pG-CX-PE and VHH-PG-PAM/pG-CX-bF-PE were unable to prevent the mammosphere formation from MCF-10A, which were transfected either at seeding or after five days of growth in mammosphere culture conditions. However, the above mentioned dendriplexes led to a reduction in MCF-10A MFE % by Day 7 (from 0.5 ± 0.11% for non-treated to 0.2 ± 0.62%, 0.3 ± 0.17%, and 0.3 ± 0.55% in case of seeding stage transfection; and to 0.3 ± 0.05%, 0.3 ± 0.62%, and 0.4 ± 0.15% in case of transfecting the existing mammospheres, respectively).

## Conclusion

In summary, we successfully developed a well-designed multi-targeted nanosystem using anti-HER2 VHH functionalized PAMAM, CXCR1 promoter, PE38 toxin and bFGF 5′UTR. This nanosystem was selectively internalized, specifically transcribed, efficiently translated and caused a selective cytotoxicity in HER2-positive BCSCs. Our data suggest that this novel multi-targeted nanosystem would be a potent strategy for selective cancer killer gene therapy. We further demonstrated that the efficacy of our targeted gene therapy was lower in spheroid models compared to monolayer cultures. Since tumor spheroid models are a more similar candidate for bulky tumor lesions and their microenvironment (in patients and/or preclinical anima models) in comparison with conventional monolayer cell cultures, it might be reasonable to anticipate lower antitumor efficacy for our nanosystem in preclinical animal models of human breast cancer in comparison with that achieved from in vitro experiments.

However, in vivo validity of the proposed idea remains to be assessed.

## Supplementary Information

**Supplementary Figure S1.**

## Data Availability

All data generated or analyzed during this study are included in this published article.
