# Supplementary material for: PE38-based gene therapy of HER2-positive breast cancer stem cells via VHH-redirected polyamidoamine dendrimers
Source: Sci Rep. 2021 Jul 30;11:15517. doi: 10.1038/s41598-021-93972-5 (PMC8324773; doi:10.1038/s41598-021-93972-5)
Supplement: Supplementary file 1 — Supplementary Figure S1. [file 41598_2021_93972_MOESM1_ESM.pdf]

PE38-based Gene Therapy of HER2-positive Breast Cancer Stem Cells via VHH-redirected Polyamidoamine Dendrimers

Cobra Moradian and Fatemeh Rahbarizadeh*

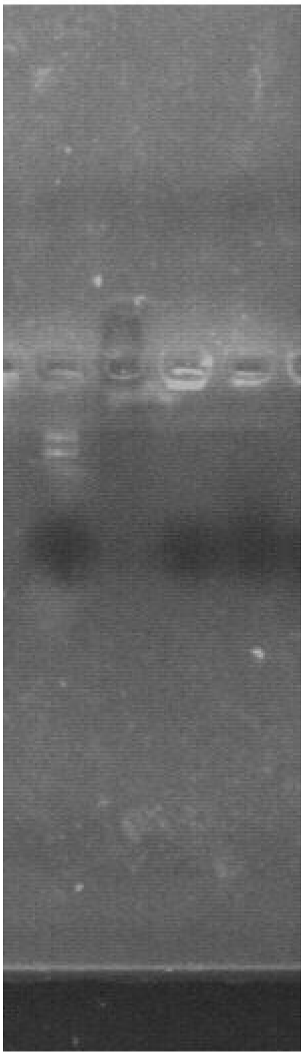

Supplementary figure 1 Plasmid mobility retardation assay by 1% agarose gel
